# Assessment of ROS1 Gene Rearrangement in Non-Small Cell Lung Cancer: Concordance Between ROS1 SP384 Immunohistochemistry and ROS1 FISH Assay in a Single Center in Türkiye

**DOI:** 10.5146/tjpath.2026.14968

**Published:** 2026-05-30

**Authors:** Claudia Andrea Gomez Gonzalez, Gurdeniz Serin, Pinar Gursoy, Ali Veral, Deniz Nart

**Affiliations:** Department of Pathology, Ege University, Faculty of Medicine, İzmir, Türkiye; Department of Medical Oncology, Ege University, Faculty of Medicine, İzmir, Türkiye

**Keywords:** Lung cancer, ROS1, SP384, FISH, IHC

## Abstract

***Objective: *** Lung cancer is the most prevalent malignancy among men and women in Türkiye. National guidelines have been established to standardize molecular pathology diagnoses for the detection of driver mutations in non-small cell lung cancer with impact in the management and treatment of the disease.

This study examines ROS1 gene alterations comparing immunohistochemistry (IHC) and fluorescence in situ hybridization (FISH) diagnostic techniques in a single-center cohort.

***Material and Methods:*** A total of 200 biopsy specimens, including tissue and cytology samples from Ege University Hospital and external consultations (2019–2022), diagnosed with non-small cell lung cancer (NSCLC) were analyzed. ROS1 SP384 IHC staining patterns (H-Score) and reflex molecular testing for EGFR, ALK, and ROS1 were recorded.

***Results: *** Among 34 ROS1 IHC-positive cases (16.9%), ROS1 FISH positivity was observed in 7 cases (3.5%), including 5 IHC-positive (5/34; 14.7%) and 2 IHC-negative (2/166; 1.2%). The median H-Score of FISH-positive cases was 60.

***Conclusion:*** ROS1 IHC exhibited staining in cases harboring ALK, EGFR, and KRAS mutations, including one case with concurrent EGFR and ROS1 alterations. The ROS1 SP384 clone demonstrated 71.43% sensitivity and 84.97% specificity, with a negative predictive value of 99.3%. ROS1 IHC is a valuable screening modality for molecular alterations. FISH validation is recommended, and discordant cases may require NGS or RT-PCR for rare mutations or unidentified fusion partners.

## INTRODUCTION

Lung cancer is a worldwide cause of death with a high-burden disease in males and females (1). In Türkiye, according to local and worldwide epidemiologic investigations, lung cancer is the most common cancer type in men and women, with a trend of increasing cases in women. Also, lung cancer is the main cause of death by cancer in Türkiye (2,3). The treatment of lung cancer is a global concern and therefore, international societies such as the International Association for the Study of Lung Cancer (IASLC), the European Society for Medical Oncology (ESMO), the American Society of Clinical Oncology (ASCO), and the National Comprehensive Cancer Network (NCCN) have developed guidelines to create a consensus and set the framework of therapeutic interventions (4–8). The pathological diagnosis with histological assessment, immunohistochemistry (IHC), and molecular studies is fundamental for the treatment. Lung cancer is mainly classified based on histopathology in non-small cell lung cancer (NSCLC), including adenocarcinomas (invasive non-mucinous, invasive mucinous, colloid, fetal, enteric-type), squamous cell carcinomas, adenosquamous carcinoma, large cell carcinoma, sarcomatoid carcinomas; and small cell lung carcinoma (SCLC).

The identification of oncogenic genomic alterations and the development of targeted therapies in NSCLC have enabled personalized treatment and improved outcomes and survival. Molecular testing for driver mutations has become an essential tool to identify the selection of patients for personalized therapy (9).

Broad molecular profiling is crucial for improving the care of patients with NSCLC. Current guidelines such as ESMO, NCCN, ASCO recommend performing molecular testing for the comprehensive predictive biomarkers by using large multigene NGS assays. Nonetheless every country has its own biomarker testing practice according to their country-specific local and regional factors and availability. In Türkiye, a molecular testing algorithm is implemented according to a regional guideline that resembles the current international guidelines (10).

Predictive immunochemistry is a timesaving, cost-effective, and feasible method in cases with limited tumor tissue and/or lack of advanced molecular techniques. The spectrum of commercial antibodies for gene assessment of predictive biomarkers in advanced NSCLC is increasing (11,12).

In the case of ROS1 gene evaluation, the FISH technique is accepted as the gold standard. Positive results on immunohistochemistry for ROS1 might have a positive correlation with FISH or not. Recent reports demonstrated positive ROS1 staining with different underlying gene mutations, such as in the EGFR, ALK, and KRAS genes, and therefore it must be confirmed by molecular techniques (13,14).

We present the status of ROS1 gene rearrangement, evaluating the immunohistochemistry technique with the ROS1 SP384 clone, and FISH technique correlation in a single center in Türkiye.

### ROS1 Gene

The ROS1 (c-Ros oncogene-1) gene is localized within chromosome 6 (6q22), a transmembrane insulin receptor family member with a tyrosine kinase domain. The breaking points of ROS1 are 32, 34, 35, and 36 exons, and the documented gene fusion partners are SCL34A2, CD74, TPM3, SDC4, EZR, LRIG3, FIG, GOPC, MSN, KDELR2, CCDC6 (15,16). The mutation frequency of ROS1 is 0.9-2.6%, observed in young, non-smoker patients, with female predominance, tumor histology with lepidic, acinar, or solid adenocarcinomas also with hepatoid features, diagnosed at an advanced stage (stage III-IV) with a higher frequency of brain metastases, and prothrombotic events (17,18).

### Assessment of ROS1 Gene Rearrangement

#### Immunohistochemistry

International guidelines suggest the use of immunohistochemistry D4D6 (Cell Signaling Technology), SP384 (Roche Tissue Diagnostics/Ventana) and clone 1A1 (Origene), as a screening tool (12). No validation of the immunostaining pattern and cut-off has been accepted yet for any commercial clone.

Descriptions of different staining patterns according to different gene partners fusion include granular cytoplasmic, globular cytoplasmic, membranous linear (apical/lateral), vesicular, and strong cytoplasmic (11,12,15).

Pneumocytes, macrophages, and giant cells show positive staining besides tumor cells, misleading false positive results (13,19,20).

The evaluation of ROS1 immunohistochemistry staining uses the H-score for D4D6 and SP384 clones, considering the scale of staining intensity (0 to 3) and the percentage of cells. In general, H-scores above or equal to 100 are considered positive and the cut-off is 150. A positive ROS1 result should be confirmed by the FISH or NGS technique (21). Due to the high sensitivity of IHC, negative results for ROS1 IHC staining can be assumed as negative for ROS1 fusion (11).

#### ROS1 Fish Technique

The ROS1 break-apart probe test is accepted as a valid test to confirm gene rearrangement, and hence to access Tyrosine Kinase Inhibitors (TKI) treatment. Analysis of at least 50 cells 15% or more of cells with rearrangement confirms the ROS1 rearrangement. A break apart pattern (green and red signals separated by a longer distance than the diameter of the largest signal pair), and isolated 3’ pattern (isolated green signal and fusion signal) are considered positive findings. A predominant pattern of isolated 5’ signal might require additional molecular tests to rule out cryptic rearrangements (21).

#### Polymerase Chain Reaction (PCR) and Next-Generation Sequencing (NGS)

Abnormal cDNA or mRNA sequences formed by rearrangement can be detected by PCR or NGS. RT-PCR assays require multiple sets of specific primers to distinguish between known fusion variants; this can be confirmed by subsequent sequencing. Therefore, RT-PCR will likely miss the rare variants, and as the list of ROS1 fusion partners is quite large and continues to grow, the use of RT-PCR is limited in clinical practice for the detection of ROS1 rearrangement. DNA-based NGS tests that sequence the intronic regions of the analyzed genes include relatively large regions of the genome. RNA-based NGS tests are specifically designed to detect gene fusions and perform well in diagnosing ROS1 gene translocation (22).

## MATERIALS and METHODS

**Figure 1:**
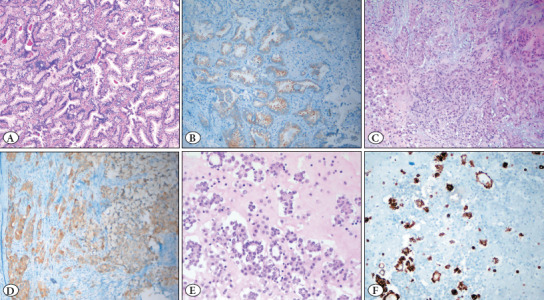
Hematoxylin-eosin staining and scoring of ROS1 IHC (SP384) 10x. **A,B)** ROS1 score 1+ (weak). **C,D)** ROS1 score 2+ (moderate). **E,F)** ROS1 score 3+ (strong)

This retrospective study included 200 biopsies performed in Ege University Hospital and consultation material referred to the center for molecular studies during the period of January 2019 to January 2022. Tissue specimens (small biopsies and resections) and cytology specimens; primary and metastatic tumors with the diagnosis of NSCLC submitted for molecular studies were included. For each case, the demographic information (age, gender, smoking history), histologic type, ROS1 immunohistochemistry, and at least reflex molecular testing for three genes (EGFR, ALK, and ROS1) were retrieved from pathology archives and the hospital’s electronic records. The follow-up information of the patients with ROS1 rearrangement was provided by the Oncology Department of Ege University Hospital. The ROS1 clone SP384 was evaluated in all cases using full-section slides to account for potential intratumoral heterogeneity. The reevaluation of staining and H-score calculation was done by D.N. and C.A.G.G., pathologists with experience in lung pathology and cytopathology. The immunohistochemical staining evaluation of the tumoral cells included the cytoplasmic staining pattern, the staining intensity, that was scored into 0, 1+, 2+, and 3+ (negative, weak to strong staining), the tumoral area (expressed in percentage), and the heterogeneity. Then H-score was calculated by the sum of the multiplication of the percentage by the staining intensity (1 × [percentage of tumor cells with 1+ staining] + 2 × [percentage of tumor cells with 2+ staining] + 3 × [percentage of tumor cells with 3+ staining]) (Figure 1).

While the standard H-score ≥ 150 was considered the clinical reference cut-off, multiple immunohistochemical thresholds were analyzed for comparative purposes to assess diagnostic performance.

The H-score of any value was contrasted with the FISH result of ROS1 (molecular test results for the ALK, EGFR, KRAS genes were also contrasted).

### ROS1 SP384 Immunohistochemical Technique

In order to obtain the immunohistochemical ROS1 slides using clone SP384 from Ventana Medical Systems, TMA sections were baked for 45 min at 72 °C. Antigen retrieval was performed using Ventana’s CC1 buffer at 100 °C for 64 min. This was followed by preprimary peroxidase inhibitor and then incubation with the primary monoclonal rabbit anti-ROS1 antibody (clone SP384, RTU, Ventana Medical Systems, Tucson, AZ, USA) at 32 °C for 20 min at the BenchMark Ultra instrument (Ventana Medical Systems Inc.) in combination with the UltraView DAB IHC Detection kit, according to the manufacturer’s instructions (19,23).

### Fluorescence in situ Hybridization for ROS1

ROS1 FISH was performed at our center with a dual break apart probe from Abbott (Vysis ROS1 Break Apart FISH Probe Kit). The preparation was following the manufacturer’s recommendations. Cases were classified as ROS1 FISH-positive if they showed 15% cells with at least one signal distance apart or isolated centromeric 30 (green signal) patterns. The 15% cut-off was determined by in-house validation that was consistent with international guidelines (21).

### Statistical Analysis

We evaluated the sensitivity and specificity of ROS1 SP384 IHC using the MedCalc Statistical Software version 14.8.1. For the analysis of independent variables, as demographic variables (age and gender), histopathological subtypes and biopsy sample type IBM SPSS statistics 27 software was used. Comparison between groups was using crosstabulation with Chi-Square and Fisher’s exact tests. A *p*-value of <0.05 was considered statistically significant.

### Ethic Approval

This investigation was submitted to the clinical ethic committee with approval of Ege University Hospital (2024-221225-2.1T/45).

## RESULTS

### Cohort Description

Our cohort included 200 patients, comprising 151 males (75.5%) and 49 females (24.5%), with a median age of 64.3 and 61.3 years, respectively.

The distribution of histological diagnoses within the cases was as follows: 90 (45%) adenocarcinoma, 8 (4%) squamous cell carcinoma, 5 (2.5%) invasive mucinous adenocarcinoma, 95 (47.5%) NSCLC (Not Otherwise Specified), 1 (0.5%) enteric-type adenocarcinoma, and 1 (0.5%) sarcomatoid carcinoma.

Forty-nine (24.5%) biopsies were cytology (cell block) samples, and 151 (75.5%) were tissue samples.

### ROS1 Status

We detected 34 cases (16.9%) with a positive result for ROS1 immunohistochemistry (IHC). The H-Score ranged between 5 and 300 (average: 80.44).

ROS1 FISH was positive in 7 cases from the cohort (3.5%). Five of these cases also showed positive ROS1 IHC staining (5/34; 14.7%), while 2 cases were negative for ROS1 IHC staining (2/166; 1.2%). The median H-score for ROS1 FISH-positive cases was 60 (range: 0–270).

The comparison between cases with ROS1 positive rearrangement and ROS1 negative rearrangement did not exhibit statistical significance in terms of gender, age, biopsy sample type, or histological subtype (Table 1).

**Table 1:** Comparison between ROS1 positive rearrangement group and ROS1 negative rearrangement group

**Variable**	**ROS1 positive rearrangement**	**ROS1 negative rearrangement**	**p value**
Age (mean, S.D.)	57.57 (11.87)	63.82 (10.05)	p>0.05
Gender, n (%) Female Male	- 1 (14.3) 6 (85.7)	- 48 (24.9) 145 (75.1)	- - p>0.05
Biopsy type, n (%) Tissue sample Cytology	- 6 (85.7) 1 (14.3)	- 145 (75.1) 48 (24.9)	- - p>0.05
Histological subtype, n (%) NSCLC Adenocarcinoma Invasive mucinous adenocarcinoma Enteric-type adenocarcinoma Squamous cell carcinoma Sarcomatoid carcinoma	- 2 (28.6) 5 (71.4) - - - -	- 93 (48.2) 85 (44) 5 (2.6) 1 (0.5) 8 (4.1) 1 (0.5)	- - - - - - p>0.05
H-score ROS1 SP384 (median, range)	60 (0-270)	0 (0-300)	**p<0.05**

**NSCLC:** Non-small cell lung carcinoma

### Evaluation of ROS1 384 IHC Results

Among the ROS1 IHC positive cases (n=34), we detected 5 cases with concordant ROS1 FISH results (5/34; 14.7%) and 29 cases with discordant ROS1 FISH results (29/34; 85.29%). (A detailed description of cases is provided in Table 2, Table 3).

**Table 2:** Description of concordant and discordant cases between ROS1 IHC and ROS1 FISH techniques

**Case number**	**Age**	**Gender**	**Smoking history**	**Biopsy specimen**	**Histological diagnosis**	**H-score ROS1 IHC**
***Cases with ROS1 IHC and ROS1 FISH concordance***						
1	60	M	90 p/y, 3.5 years ex-smoker.	Lung wedge biopsy	Adenocarcinoma, lepidic predominant (70% lepidic, 30% acinar)	60
2	46	F	Unknown	Fine needle aspiration pleura	Lung adenocarcinoma metastasis, cribriform predominant (%80 cribriform, %20 acinar)	270
3§	78	M	15 p/y, 25 years ex-smoker.	Lung wedge biopsy	Adenocarcinoma, lepidic predominant (60% lepidic, 40% acinar)	210**
4	53	M	Unknown	Lymph node biopsy	Lung adenocarcinoma metastasis	160
5	58	M	Unknown	Lobectomy	Adenocarcinoma	60
***Cases with ROS1 IHC and ROS1 FISH discordance***						
1	65	M	Unknown	Liver biopsy	Lung adenocarcinoma metastasis	0
2	43	M	Unknown	Lobectomy	Adenocarcinoma	0

**p/y:** pack-year index §Case 3 had ROS1 FISH positive rearrangement and EGFR PCR exon 19 224del15. Detailed H-score 60% 3+ and 30% 1+

**Table 3:** Discordant cases of positive ROS1 immunohistochemistry with other oncogenic drivers positive

**Case number**	**Age**	**Gender**	**Smoking history**	**Biopsy specimen**	**Histology**	**H-Score**	**Oncogenic mutation**
6	55	M	60 y/p, active smoker	Lobectomy	Adenocarcinoma, acinar predominant (60% acinar + cribriform 30% + micropapillary 10%).	40 (20% 2+)	ALK
7	59	F	Unknown	Brain resection	Lung NSCLC metastasis	270 (90% 3+)	ALK
8	82	F	Unknown	Lung needle core biopsy	Lung NSCLC	20 (10% 2+)	EGFR (Codon 861 CTG>CAG mutation)
9	59	F	Unknown	Lung wedge biopsy	Adenocarcinoma (lepidic pattern)	10 (10% 1+)	EGFR (Exon 19 2235del15)
10	61	M	34 p/y	Lung wedge biopsy	Adenocarcinoma acinar predominant (acinar 40%, micropapillary 30%, lepidic 30%)	10 (10% 1+)	EGFR (Exon 19’ del).
11	76	M	Unknown	Lung needle core biopsy	Adenocarcinoma	120 (60% 2+)	EGFR (Exon 19’ 2236del15 )
12	60	F	Unknown	Lung fine needle aspiration	Lung NSCLC, adenocarcinoma	40 (40% 1+)	EGFR (Exon 19’ 2235del15)
13	77	M	Unknown	Lung wedge biopsy	Adenocarcinoma lepidic predominant (lepidic 60%, 20% acinar, 20% solid)	20 (20% 1+)	KRAS (Exon 2 codon 12 GGT> TGT mutation)

**p/y:** pack-year index

Evaluation of the discordant cases revealed the following: ALK break apart was detected in 2 cases (2/34; 5.8%) with an average H-score of 155.

**Figure 2:**
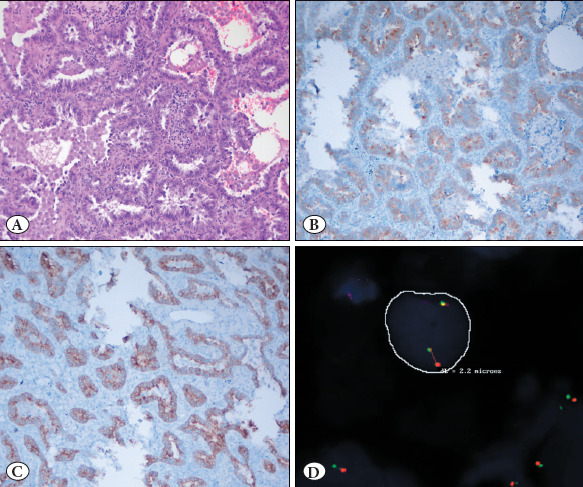
Histopathological findings of Case 3. ROS1 rearrangement and EGFR mutation. **A)** Hematoxylin-eosin staining. **B)** Tumoral areas showing ROS1 IHC positivity 1+ score. **C)** Tumoral areas showing ROS1 IHC +3 score. **D)** ROS1 FISH break apart probe positive.

EGFR mutation was found in 6 cases (6/34; 17.64%), exhibiting mutational signatures of exon 19 deletions and codon 861 mutations, with an average H-score of 68. One of these cases had concurrent ROS1 FISH rearrangement and an EGFR mutation (Exon 19 224del15) (1/34; 2.94%) with an H-score of 210 (Figure 2).

KRAS gene mutation was observed in one case (1/34; 2.94%) with a mutational signature of exon 2 codon 12 GGT>TGT and an H-score of 20.

No mutation was found in 21 cases (21/34; 61.76%), with an average H-score of 68.80.

**Figure 3:**
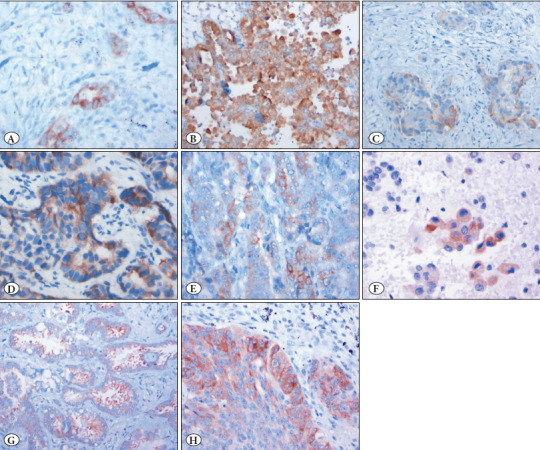
Staining pattern in cases with driver mutations in genes other than ROS1. **A,B)** ALK positive, **C-G)** EGFR positive, **H)** KRAS positive.

The staining patterns of the cases with driver mutations in genes other than ROS1 are shown in Figure 3.

### ROS1 384 Diagnostic Test Accuracy

We performed the diagnostic test accuracy evaluation for ROS1 SP384 using three distinct H-score restrictions:

All H-scores >0 were included: This yielded a sensitivity of 71.43% (95% CI: 29.04% to 96.33%) and a specificity of 84.97% (95% CI: 79.14% to 89.70%), with a Negative Predictive Value (NPV) of 99.3% (95% CI: 97.83% to 99.79%).

Restriction applied for H-scores ≥150: The sensitivity and specificity values were 42.86% (95% CI: 9.9% to 81.59%) and 97.41% (95% CI: 94.06% to 99.15%), respectively, and the NPV was 98.82% (95% CI: 97.78% to 99.37%).

H-scores with 2+ or above staining intensity in >30% of total tumor cells: The sensitivity and specificity values were 71.43% (95% CI: 29.04% to 96.33%) and 100% (95% CI: 98.11% to 100%), respectively, and the NPV was 99.42% (95% CI: 98.15% to 99.82%) (Table 4).

**Table 4:** Diagnostic test evaluation results for ROS1 SP384 IHC

**Statistics (value, 95%CI)**	**H-score>0**	**H-score>150**	**H-score 2+ or above staining intensity in more than 30% of total tumor cells**
Sensitivity	71.43% (29.04% to 96.33%)	42.86% (9.90% to 81.59%)	71.43% (29.04% to 96.33%)
Specificity	84.97% (79.14% to 89.70%)	97.41% (94.06% to 99.15%)	100% (98.11% to 100%)
Positive Predictive Value	8.84% (5.17% to 14.72%)	25.24% (9.09% to 53.26%)	100% (47.82% to 100%)
Negative Predictive Value	99.32% (97.83% to 99.79%)	98.82% (97.78% to 99.37%)	99.42% (98.15% to 99.82%)
Accuracy	84.70% (78.95% to 89.39%)	96.32% (92.68% to 98.47%)	99.43% (97.13% to 99.98%)

The clinical follow-up of the seven patients with genetic mutations was as follows: Only one patient received tyrosine kinase inhibitors in conjunction with adjuvant chemotherapy; this patient unfortunately died during therapy. Three patients received adjuvant chemotherapy; two of these patients died within a short follow-up period, and one patient is still alive but is currently receiving treatment at another hospital.

## DISCUSSION

In our cohort, the prevalence of ROS1 rearrangement was 3.48%, which is higher than the average reported prevalence of 0.9–2.6% in the literature (18). However, Prall et al. (24) reported a higher prevalence of 4.6% for ROS1 rearrangements in their cohort.

### ROS1 Immunohistochemistry Assessment 

Assessment of ROS1 can be performed by immunohistochemistry (IHC) using commercial antibodies, primarily D4D6 and SP384. Most previous studies have employed the D4D6 antibody, reporting sensitivities ranging from 94.4% to 100% and specificities from 63.6% to 96.9% (25). Across the literature, the use of ROS1 SP384 as a screening technique for ROS1 rearrangement has recently been increasing (26–28).

Several cut-offs have been proposed for interpreting ROS1 IHC results: Conde et al. (29) suggested a positive cut-off of H-score ≥150 or ≥70% of tumor cells with moderate (2+) or stronger staining intensity. Huang et al. (30) compared ROS1 IHC and FISH, defining an IHC-positive result as cytoplasmic staining of 2+ or above intensity in more than 30% of total tumor cells, and reporting a sensitivity of 97.8% and specificity of 89.5%. Prall et al. (24) proposed evaluating ROS1 IHC based only on the percentage of tumor cells with positive staining, regardless of intensity, establishing a cut-off of 50% or more for cases requiring FISH confirmation. Ahn et al. (31) suggested using an H-score cut-off of 287.5 and evaluating the homogeneity/heterogeneity of the staining pattern to improve sensitivity, considering a diffuse homogeneous pattern (≥2+ staining in all tumor cells) as a positive result. Dyrbekk et al. (27) also noted that a homogeneous staining pattern was consistent with ROS1 rearrangement.It is important to note that while an H-score ≥150 remains the established reference in routine clinical practice, exploring multiple immunohistochemical thresholds is essential for research purposes to assess and potentially optimize diagnostic performance in different settings.

In our study, the following results were obtained based on different criteria:

Initial evaluation (Any staining ≥1+, regardless of percentage): Sensitivity was 71.43% and specificity was 85.05%.

Restriction to an H-score ≥150: Sensitivity was 42.86% and specificity was 97.42%.

Applying the criteria suggested by Huang et al. (30)[ƒ: Sensitivity was 71.43% and specificity was 92.27%.

Therefore, we agree with the criteria suggested by Huang et al. (30) for the assessment of IHC, acknowledging that while different from the routine H-score ≥150, they provided a better balance of sensitivity and specificity in our specific cohort.

### Co-mutations and Clinical Implications

We detected one case of ROS1 rearrangement with a synchronous EGFR Exon 19 224del15 mutation. Oncogenic ROS1 co-mutations have been reported in up to 36% of cases, primarily with KRAS, EGFR, and ALK mutations (32). Crosstalk between the ROS1 and EGFR pathways is recognized (20). ROS1 co-mutation has been reported as a resistance mechanism to EGFR-tyrosine kinase inhibitors (TKIs) in the presence of EGFR mutation signatures like exon 19 deletion, exon 20 insertions, or L858R mutations (33,34). The clinical implications of double or triple-positive oncogenic drivers can be challenging when selecting treatment regimens, thus impacting patients’ clinical outcomes.

### IHC Positivity in ROS1-Negative Cases

ROS1 IHC staining can be observed in cases with driver mutations in genes other than ROS1. In our cohort, this phenomenon was noted in cases with EGFR, ALK, and KRAS mutation signatures. Similar observations have been reported in the literature for driver mutations involving RET, EGFR, ALK, and MET genes, utilizing both the D4D6 and SP384 commercial clones (20,26,27,35,36). The pathogenicity of the ROS1 gene in lung cancer is defined by its rearrangement, although copy number alterations have also been recognized without impact on patient outcomes (37). Grenier et al. (20) evaluated ROS1 (D4D6) IHC results against ROS1 mRNA transcript levels and found that IHC positivity is consistently related to true ROS1 gene expression. They also noted an association between ROS1 IHC positivity and cases with EGFR mutations, with a similar tendency observed for MET mutations. Although the precise molecular mechanism remains unknown, authors suggest a possible explanation is that ROS1 expression might be increased in response to specific oncogenic driver activation, implying a pleiotropic role in NSCLC independent of ROS1 rearrangements.

### Discordant Cases

The 29 discordant cases (IHC positive but molecularly negative) in our cohort, which lacked a molecularly positive finding, may be due to the presence of another oncogenic driver mutation or a false-negative FISH result for fusion partners such as GOPC–ROS1 or EZR–ROS1 (38,39). Furthermore, rare mutations and amplifications of ROS1, such as SQSTM1, have been reported.A significant limitation of this study is the lack of confirmation via RNA-based Next-Generation Sequencing (NGS) or RT-PCR in these discordant cases. Such molecular methods are considered the gold standard for resolving discrepancies between IHC and FISH, as they can identify specific fusion transcripts and confirm true gene expression. Therefore, the application of RT-PCR or Next-Generation Sequencing (NGS) is suggested for definitive diagnosis in discordant cases and should be integrated into future research to strengthen the diagnostic algorithm.

## CONCLUSION

The assessment of driver gene mutations and the subsequent application of targeted therapies in lung cancer are constrained by international and local guidelines. Furthermore, the availability of economic resources, access to complex molecular techniques in healthcare centers, and public health policies all play a significant role in their clinical implementation.

ROS1 immunohistochemistry (IHC) serves as a helpful screening tool in lung cancer to identify potential molecular alterations in positive cases, suggesting the possibility of a ROS1 rearrangement and/or other oncogenic driver mutations. Conversely, negative IHC results are generally accepted as strong evidence for the absence of gene rearrangement.

Positive IHC results require confirmatory evaluation using either the Fluorescence In Situ Hybridization (FISH) technique or a broader approach such as RNA/DNA-based Next-Generation Sequencing (NGS).

In discordant cases—where IHC staining is positive, but FISH (or another primary molecular assay) reveals no ROS1 rearrangement, and there is no evidence of ALK, KRAS, or EGFR driver mutations—further testing is warranted. NGS or Reverse Transcription-Polymerase Chain Reaction (RT-PCR) for ROS1 should be performed to rule out rare mutations or undetected fusion partners that may be missed by the FISH technique.

Finally, the presence of ROS1 rearrangement alongside co-mutations with EGFR, KRAS, or ALK creates a therapeutic challenge for optimal medical treatment selection.

## Conflict of Interest

The authors report there are no competing interests to declare.
